# MicroRNA let-7b inhibits keratinocyte differentiation by targeting IL-6 mediated ERK signaling in psoriasis

**DOI:** 10.1186/s12964-018-0271-9

**Published:** 2018-09-15

**Authors:** Yan Wu, Liu Liu, Chunxiang Bian, Qingchun Diao, Muhammad Farrukh Nisar, Xuemei Jiang, Jörg W. Bartsch, Maojiao Zhong, Xiangyu Hu, Julia Li Zhong

**Affiliations:** 1 The Base of “111 Project” for Biomechanics & Tissue Repair Engineering, Key Laboratory of Biorheological Science and Technology, Ministry of Education, college of Bioengineering, Chongqing University, Chongqing, 400044 China; 2 Department of Dermatology, Chongqing First People’s Hospital and Chongqing Traditional Chinese Medicine Hospital, No. 40 Daomenkou St., District Yuzhong, Chongqing, 400011 China; 3 Philipps University Marburg, Department of Neurosurgery, Baldingerstr, 35033 Marburg, Germany; 4 Interdisciplinary Research Center in Biomedical Materials (IRCBM), COMSATS University Islamabad, Lahore Campus, Lahore, 54000 Pakistan

**Keywords:** Psoriasis, Let-7b, IL-6, ERK1/2, Differentiation

## Abstract

**Background:**

The extensive involvement of microRNA (miRNA) in the pathophysiology of psoriasis is well documented. However, in order for this information to be useful in therapeutic manipulation of miRNA levels, it is essential that detailed functional mechanisms are elucidated. This study aimed to explore the effects of IL-6 targeting by let-7b and ERK1/2 mediated signaling on keratinocyte differentiation in psoriasis.

**Methods:**

Following imiquimod cream (IMQ) application to let-7b^TG^ (keratinocyte-specific let-7b overexpression mouse) and control mice for 7 days, we analyzed erythema, scaling and thickening of skin. A dual luciferase reporter assay and bioinformatics was carried out to detect target gene of let-7b. Additionally, the differentiation markers were measured. Immunohistochemistry analyses demonstrate a relationship of let-7b with IL-6 and ERK signaling.

**Results:**

we found let-7b^TG^ inhibits acanthosis and reduces the disease severity by treatment with IMQ compared to wild-type mice. Further study illustrated that let-7b promotes differentiation of keratinocytes in vivo and in vitro. Using bioinformatics and reporter gene assays, we found that IL-6 is a target gene of let-7b. In psoriasis, high expression levels of IL-6 lead to increased acivation of p-ERK1/2. High levels of let-7b^TG^ transgene expression suppresses IL-6 expression and leads to increased keratinocyte differentiation. Moreover, let-7b acts as an upstream negative regulator of the ERK signaling pathway in keratinocytes of psoriasis.

**Conclusions:**

Our result reveals a previously unknown mechanism for regulation of IL-6 levels during psoriasis by let-7b and highlights a critical role for the ERK1/2 signaling pathway in epidermal differentiation during psoriasis.

**Trial registration:**

The ethical approval for this study was from the Affiliated Hospital of Medical University of Anhui _ Fast_ PJ2017–11–14.

**Electronic supplementary material:**

The online version of this article (10.1186/s12964-018-0271-9) contains supplementary material, which is available to authorized users.

## Background

Psoriasis is a long-lasting autoimmune disease characterized by patches of abnormal skin that affects 2–3% of the world’s population [1]. Psoriasis undergoes three different processes of cellular alteration in skin: abnormal differentiation of keratinocytes, hyperproliferation of keratinocytes, followed by infiltration of immune cells into the dermis and epidermis [2]. Some common molecular components, genetic alterations of genes that participate in inflammatory pathways, and environmental risks can contribute to the pathogenesis of psoriasis [3]. However, the underlying mechanisms regulating these epidermal defects and immunological dysfunction remain largely unknown.

Results from recent studies have indicated that microRNAs (miRNAs), as a potential regulator of gene expression, play important roles in psoriasis [4]. miRNAs are small endogenous RNA molecules with 22–24 bases long, which are often expressed in cell lineages and play critical roles in nearly all biological processes, including cell differentiation, development, and metabolism, as well as complex diseases [5]. Previous studies have identified a distinct miRNA expression profile in psoriasis skin compared to healthy skin [6]. Several of these deregulated miRNAs have been shown to regulate keratinocyte proliferation and differentiation, such as miR-21 [4], miR-217 [7], miR-194 [8], miR-99a [9] and miR-330 [10]. Although a large number of miRNAs have been identified, their roles in the biology of psoriasis and the underlying mechanisms have not been fully explored.

The miRNA let-7 is the first known human miRNA and second found in *Coenorhabditis elegans* (*C. elegans*) [11]. Let-7b, a member of the let-7 family, has been shown to play a role in proliferation and differentiation of neural stem cells [12], and also suppresses the odonto/osteogenic differentiation capacity of stem cells from apical papilla by targeting MMP1 [13]. Recent study demonstrated that let-7b stimulates differentiation and prevents metaplasia by regulating HNF6 expression in pancreatic acinar cells [14]. It is also becoming increasingly recognized that let-7b plays an important role in hair growth [15, 16], cutaneous wound healing [17] and skin disease [18]. Recently, it was found that let-7b inhibits keratinocyte migration through targeting IGF2BP2 [17]. As well as, let-7b has been also implicated in other malignacies e.g. liver and thyroid cancer and it exerts a tumorsuppressive role by inhibiting proliferation [19, 20]. However, the biological role of let-7b in the pathogenesis of psoriasis, and especially with regard to its influence on keratinocyte differentiation, is not fully understood.

In this study, we aimed to investigate the biological role of let-7b and the underlying molecular mechanism in regulating the pathogenesis of psoriasis. We found the expression of let-7b was markedly down-regulated in psoriasis keratinocytes in a psoriatic mouse model. We demonstrate a previously unrecognized role of let-7b in regulating the keratinocyte differentiation by using a keratinocyte-specific let-7b expression mouse model (let-7b^TG^). We have revealed that the let-7b overexpression in basal keratinocytes inhibits acanthosis and reduces the disease severity in two mouse models of psoriasis. Moreover, interleukin (IL)-6 was identified as a direct target of let-7b by which let-7b regulates keratinocyte differentiation. Taken together, our study demonstrates that let-7b regulates keratinocyte differentiation through targeting IL-6-dependent ERK1/2 signaling that may play an important role in the pathogenesis of psoriasis.

## Methods

### Specimen collection

Four patients with psoriasis and four healthy subjects were enrolled in the present study. Psoriasis patients were recruited from the Department of Dermatology, Chongqing First People’s Hospital (Chongqing, China). The psoriasis patients enrolled in this study had neither received any local treatment for two weeks nor had used any systemically immunosuppressive medications for at least one month before study participation. Punch biopsies (4 mm) were collected from psoriasis patients at the lesional site. Punch biopsies collected from the healthy subjects were used as control. The specimens were immediately frozen in liquid nitrogen and stored at − 80 °C for further use. The present study was reviewed and approved by Institutional Human Experiment and Ethic Committee of the Affiliated Hospital of Medical University of Anhui. The written informed consents were obtained from all participants.

### Animal experiment

Keratinocyte-specific let-7b expressing transgenic mice (let-7b^TG^) were kindly provided by Dr. Xiao Yang (Institute of Biotechnology, The Academy of Military Medical Sciences, Beijing, China). Because male mice are aggressive and easy to fight each other and torn the back skin. Keeping in view this aspect, we decided to choose only female mice (8–12 weeks of age) as the experimental model [21]. IMQ-induced mouse model of psoriasis: The mice were applied a daily topical dose of 62.5 mg of IMQ cream (5%) (MedShine, #120503; China) on the shaved back for seven consecutive days. At the days indicated, skin thickness was measured using a micrometer (Mitutoyo). Control mice were treated with a same dose of vehicle cream. PD98059 treatment in vivo: when mice treated with IMQ after 4 h, a daily topical dose of PD98059 (5 mg/kg, APExBIO) dissolve the 10% dimethylsulfoxide (DMSO) or DMSO directly drop on the skin to treatment psoriasis. All animal procedures were performed under the ethical guidelines of Laboratory Animal Welfare and Ethics Committee Of the Third Military Medical University and according to the recommendations of the Chongqing Experimental Animal Regulation Board (SCXK-PLA-20140011); the approval number is SYXK-PLA-20140031.

### Cell culture and transfection

HaCaT cells were cultured in Dulbecco’s Modified Eagle Medium (DMEM) supplemented with 1% Non-Essential Amino acid and 10% fetal bovine serum (Hyclone) under 5% CO_2_ at 37 °C. Transfection of HaCaT cells were done with let-7b mimic, scrambled miRNA (control group), let-7b antisense oligonucleotides or scrambled oligonucleotides (sham control group) transiently (Genepharm) with lipofectamine 2000 reagent (Invitrogen).

### Luciferase reporter assays

Firefly luciferase reporter plasmids containing 3′ -UTR of the human *IL6* gene and empty luciferase vectors were obtained from Promega. HaCaT cells were co-transfected with the luciferase reporters (50 ng per well) together with 10 nM let-7b mimic or scrambled miRNA using Lipofectamine 2000 (Invitrogen). Luciferase activity was analyzed 48 h post-transfection using Dual-Luciferase Reporter Assay System (Berthold Centro) following the manufacturer’s instructions.

### Immunohistochemistry analyses

Mouse skin tissues were embedded in paraffin, 6 μm sections were prepared and stained with K14 antibody (1:1000, Covance), IL-6 antibody (1:250, Cell Signaling Technology) and p-ERK1/2 antibody (1:250, Cell Signaling Technology) analyzed by immunohistochemistry methods. Signals were detected by DAB (Zhongshan) staining. Slides were observed under a EVOS FL Auto Microscope (Thermo Fisher).

### Culture of keratinocytes

Primary mouse keratinocytes were isolated from normal and transgenic newborn mice skin. 5 mg/ml Dispase (Invitrogen) solution was applied to the full thickness skin overnight (14–16 h) at 4 °C. The epidermis was softly separated from the dermis and transferred onto the surface of the drop of TrypLE Select with the basal layer downward (Invitrogen). The epidermis was incubated for 20–30 min at room temperature, followed by suspension in Keratinocyte-SFM medium with supplements (Invitrogen). Cells were separated from the epidermis and incubated in dishes coated with a solution containing 1% *v*/v Invitrogen 100 collagen, 10 mM HEPES, 10 μg /ml fibronectin, and 100 μg /ml BSA, at 34 °C in 5% CO_2_ [22]. Cells were further cultured in fresh Keratinocyte-SFM medium with supplements and 5% fetal bovine serum (Hyclone), which was replaced once every two days.

### RNA extraction and qRT-PCR

Total cellular RNA was extracted using TRIzol reagent (TaKaRa) following the manufacturer’s protocol. RNA was reversed transcribed to cDNA using SuperScriptIII (Promega). qRT-PCR was carried out in 20 μl volumes containing 1× Eva Green qPCR master mix (Applied Biological Materials Inc., Richmond, BC, Canada) on CFX Connect™ Real-Time PCR Detection System (USA). Primers are listed in Supplement Table S1. Glyceraldehyde-3-phosphate dehydrogenase (GAPDH) was used as a reference gene. The relative expression levels of mRNA were quantified using the ΔΔCt method.

### Western blot analysis

Western blotting was performed as follows: the protein lysates were collected from the cells. The tissue lysates were separated on 12% SDS-polyacrylamide gels and transferred onto polyvinylidene fluoride membranes (Bio-Rad). Antibodies used included CK10, CK1 (1:1000, Abcam), Involucrin, IL-6, p-ERK and ERK antibody (1:1000, Cell Signaling Technology)), while GAPDH (1:2000) was used as a loading control. The Western blot results were further analyzed using BIO-RAD ChenmiDoc™ XRS+.

### Statistical analysis

All values were expressed as mean ± S.D. Statistical analysis of the data was performed by two-tailed Student’s t test (*, *p* < 0.05; **, *p* < 0.01).

## Results

### Reduction of disease severity in let-7b^TG^ mice treated with IMQ

**Fig. 1:**
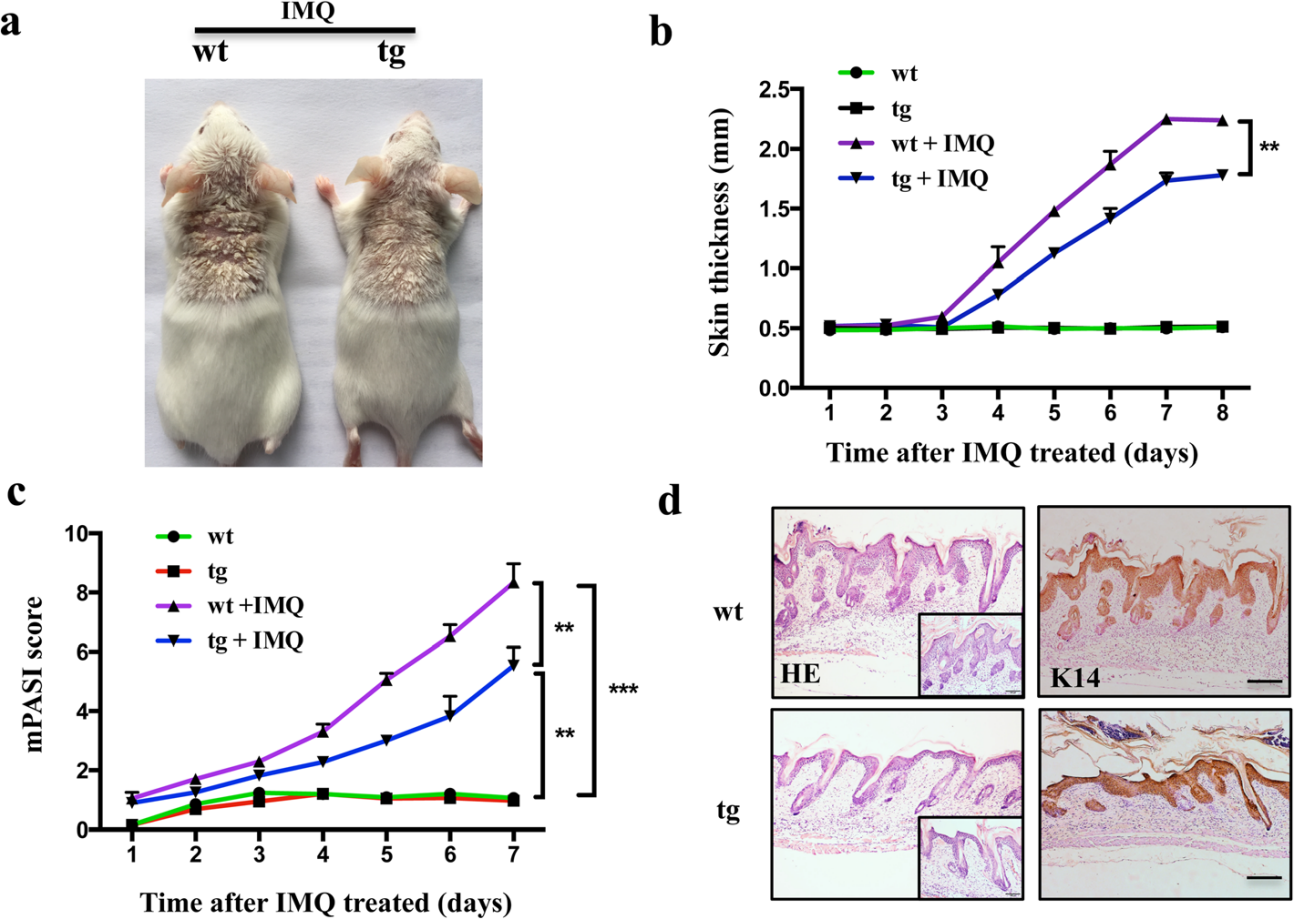
Inhibition of acanthosis and reduction of disease severity in let-7b^TG^ mice treated with IMQ. **a**. Phenotypic presentation of mouse back skin for wild-type and transgenic mice treated with IMQ for 7 days. **b**. Skin thickness was measured on the days indicated in the epidermis of wild-type and transgenic mice treated with IMQ or vehicle. Symbols represent mean skin thickness ± s.e.m. for five to six mice per group. **c** mPASI reflecting the intensity of skin inflammation. Two-tailed unpaired Student’s t-test was performed for statistical analysis. Data are shown as means ± s.e.m. **d** Light microscopy examination of skin sections stained with H&E, or with keratin K14 antibody. Scale bar, 200 μm. Scale bar in right image below, 100 μm

Our previous study revealed that let-7b inhibits re-epithelialization in wound healing by targeting IGF2BP2. In the course of this study, a transgenic mice with keratinocyte-specific let-7b overexpression (let-7b^TG^) in skin with K5 promoter was generated [17]. In our study, real-time PCR analyses showed transgenic mice overexpressed let-7b efficiently in skin tissues, especially in primary keratinocyte (Additional file 1: Figure S1a). Hence, these data confirmed the skin of transgenic mice have stable overexpress let-7b. It is noteworthy that the wound healing process and the pathogenesis of psoriasis have similarities, especially the inflammatory response, keratinocyte proliferation, as well as vascular endothelial cell proliferation and differentiation [23]. To elucidate the potential role of let-7b in skin psoriasis, we topically applied imiquimod cream containing 5% IMQ, on the shaved back skin of let-7b^TG^ and wild-type mice for 7 consecutive days to induce psoriasis. In agreement with another study [21], both let-7b^TG^ and wild-type mice treated with IMQ developed sharply demarcated erythematous lesions covered with white silvery squama (Fig. 1a). Imiquimod cream application onto the skin of let-7b^TG^ mice for 7 days reduced skin inflammation reflected by erythema, scaling and thickening (Fig. 1a). Strikingly, we demonstrated that a let-7b-specific overexpression in the epidermis resulted in a pronounced decrease in skin thickness in let-7b^TG^ mice treated with IMQ (Fig. 1b). Moreover, compared with IMQ-treated wild-type (WT) and to mice treated with an emollient cream as control, let-7b^TG^ mice showed a striking decrease of skin inflammation as reflected by an increased modified Psoriasis Area and Severity Index (mPASI) which was adapted to mice pathology (Fig. 1c). Microscopic examination of haematoxylin and eosin-stained skin sections from IMQ-treated let-7b^TG^ mice revealed many histological features of chronic inflammation that are characteristic for human psoriasis including parakeratosis, acanthosis and elongation of the dermal papillae. Immunohistochemistry showed an increased expression of keratin K14 in suprabasal epidermal layers indicated an accelerated keratinocyte proliferation (Fig. 1d). These data imply that intrinsic let-7b plays a pivotal role in psoriasis.

### Accelerated cell differentiation in let-7b^TG^ mice

**Fig. 2:**
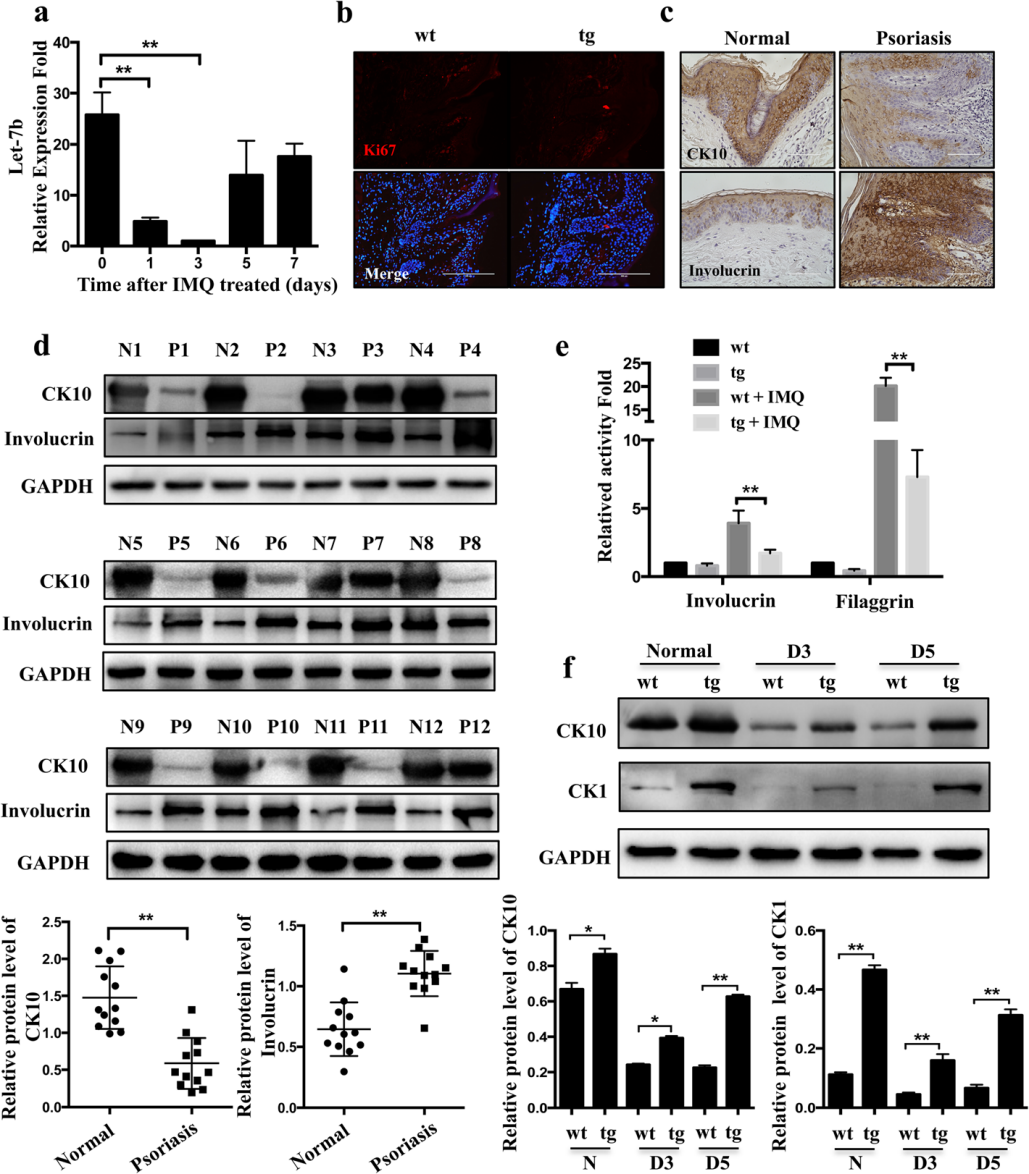
Let-7b promotes differentiation of keratinocytes in the let-7b^TG^ mouse model. **a** Let-7b expression was detected by real-time PCR in wild-type mice treated with IMQ for 0, 1, 3, 5, 7 days. **b** Immunofluorescence staining with Ki67 (red) antibody was carried out at day 5 after treated with IMQ in wild-type and transgenic mice. Nuclei were stained with DAPI (blue signal). No noticeable difference was found in percentage of Ki67 positive keratinocytes in wild-type and transgenic mice. Scale Bar = 200 μm. **c** Immunohistochemistry analysis the expression of CK10 and involucrin in normal subjects (left) and psoriatic patients (right). scale Bar = 100 μm. **d** Western blot analysis of epidermal differentiation markers (Keratin 10 and Involucrin) in the epidermis of normal skin derived from twelve healthy individuals (N1 to 12) or in psoriatic lesions derived from twelve patients with psoriasis (P1 to 12). The columnar was relative quantitation of CK10 and involucrin protein levels in twelve different samples, which were shown after normalization to the endogenous control of GAPDH (Mean ± SD). **P<0.01. **e** Real-time PCR analysis of Involucrin and Filaggrin expression in the epidermis of wild-type and transgenic mice treated with IMQ or vehicle. **f** Western blot analysis of epidermal differentiation markers (Keratin 10 and K1) in wild-type and transgenic mice treated with IMQ for 0, 3, 5 days. The columnar was relative quantitation of CK10 and involucrin protein levels, which were shown after normalization to the endogenous control of GAPDH (Mean ± SD). *P<0.05; **P<0.01.

To further investigate the potential role of let-7b in the aetiology and pathogenesis of psoriasis, we compared let-7b expression between normal and psoriasis skin of wild-type mice. Real-time PCR quantified the total RNA level of let-7b in IMQ-treated wild-type mice, which was significantly decreased by 75% and 90% at days 1 and 5 after treatment with IMQ (Fig. 2a, *P* < 0.01). Psoriasis is usually characterized by hyperproliferation and abnormal differentiation of keratinocytes [24]. In the previous study, we found that let-7b had no effect on cell proliferation on wound healing [17]. Now our study again illustrated the let-7b had no relationship with proliferation in psoriasis (Fig. 2b), so we focus on the differentiation function of let-7b in psoriasis. In normal skin, differentiation marker involucrin was exclusively detected in the keratinocyte and upper stratum spinosum and CK10 was expression widely. In contrast, in psoriatic lesional skin, involucrin was identified in almost the whole stratum spinosum while CK10 expression decreased and alsmost in keratinocyte (Fig. 2c). Then, we examined the differentiation markers expression by western blot in full- depth biopsies of lesional skin from psoriasis patients (*n* = 12) and healthy skin (n = 12), the results showed an increase in the numbers of late differentiation markers Involucrin and a decrease in K10-expressing cells in the epidermal layer reflecting abnormal differentiation in psoriasis skin lesions compared to healthy skin (Fig. 2d). To determine whether let-7b in psoriasis keratinocyte affects its differentiation and lead to reduce disease severity, we analyzed the differentiation status of keratinocytes at day 3 by Real-time PCR after treatment with IMQ. The results show that the expression of Involucrin and Filaggrin in the epidermis of let-7b^TG^ mice are not significantly different in normal skin, whereas we observed decreased expression of let-7b in the IMQ-induced psoriasis mouse model (Fig. 2e). We further examined the expression of early differentiation markers such as cytokeratin 10 (CK10) and cytokeratin 1 (CK1) from healthy skin and the psoriasis lesion in transgenic mice and found it markedly increased compared to that of control littermates (Fig. 2f). Collectively, our data indicate that let-7b promotes keratinocyte differentiation and lead to a reduced disease severity.

### Let-7b promotes keratinocyte differentiation in HaCaT

**Fig. 3:**
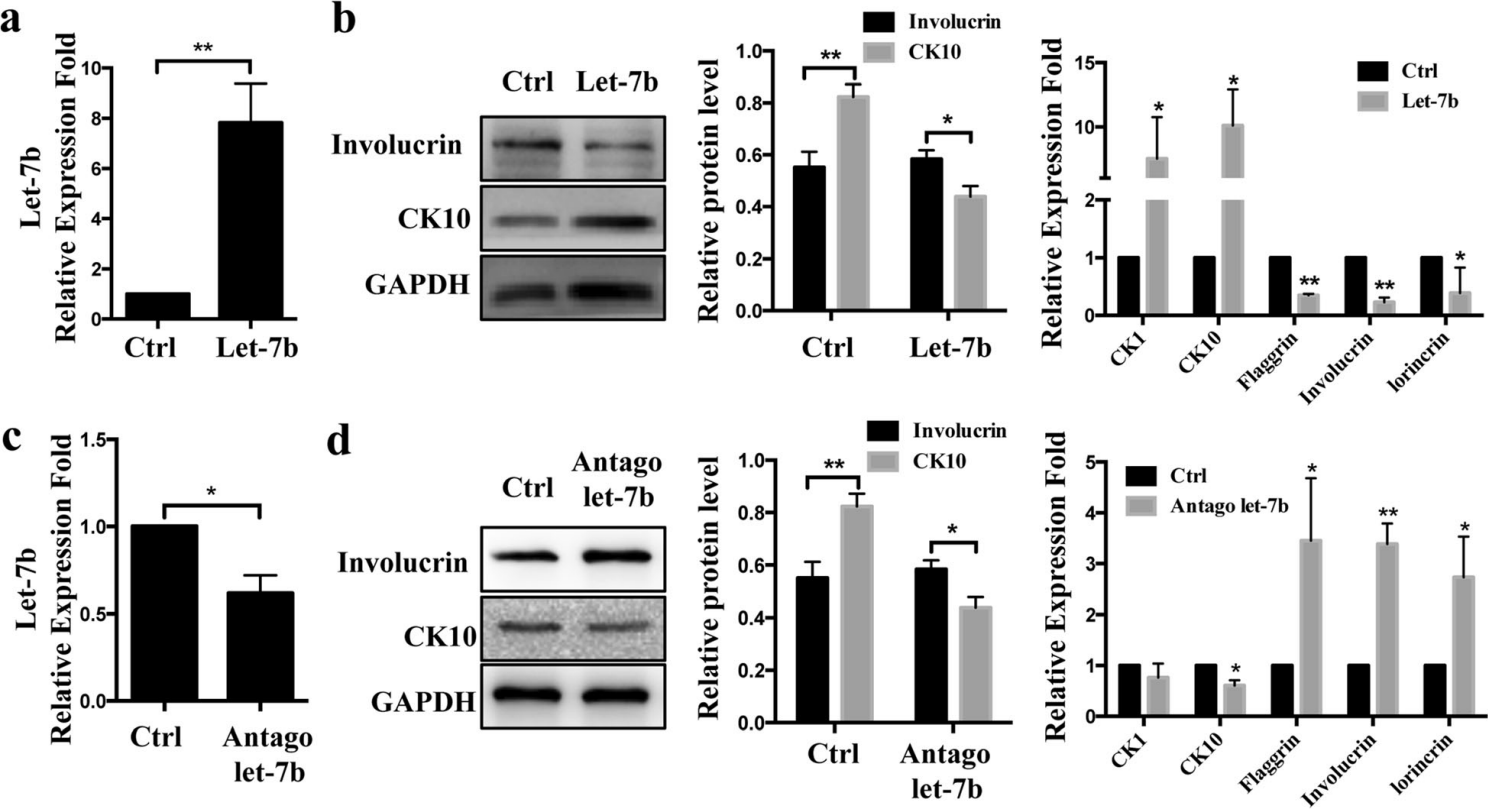
Let-7b promotes cell differentiation in HaCaT. **a** Overexpression of let-7b in HaCaT transfected with let-7b mimic compared to HaCaT transfected with let-7b negative control (miR-NC). **b** Western blot and Real-time PCR analysis of early and late epidermal differentiation markers cytokeratin 1 (CK1), cytokeratin 10 (CK10), filaggrin, Involucrin and Loricrin. **c** HaCaT cells were transfected with let-7b inhibitor and inhibitor negative control (“InNC”). **d** Western blot and Real-time PCR analysis of early and late epidermal differentiation markers. Data are shown as mean ± SD from three independent experiments. *P < 0.05, **P < 0.01

To further investigate if the role of let-7b in vitro is coherent with the in vivo situation, we analyzed the differentiation status of HaCaT after transfection with a let-7b mimic or an inhibitor as well as their respective controls. We measured the expression of both early and late differentiation markers such as cytokeratin 1 (CK1), cytokeratin 10 (CK10) and Involucrin, filaggrin, Loricrin (Fig. 3a). Overexpression of let-7b significantly increased CK10 and decreased Involucrin expression at protein levels, whereas inhibition of endogenous let-7b decreased CK10 and increased Involucrin expression levels (Fig. 3c). Similar results were obtained for mRNA expression levels of five differentiation markers CK1, CK10, Involucrin, Flaggrin and lorincrin in keratinocytes with overexpression or inhibited let-7b (Fig. 3b and d). These results suggest that let-7b affects the differentiation of HaCaT cells.

### The 3’ UTR of the *IL-6* gene is a target of let-7b

**Fig. 4:**
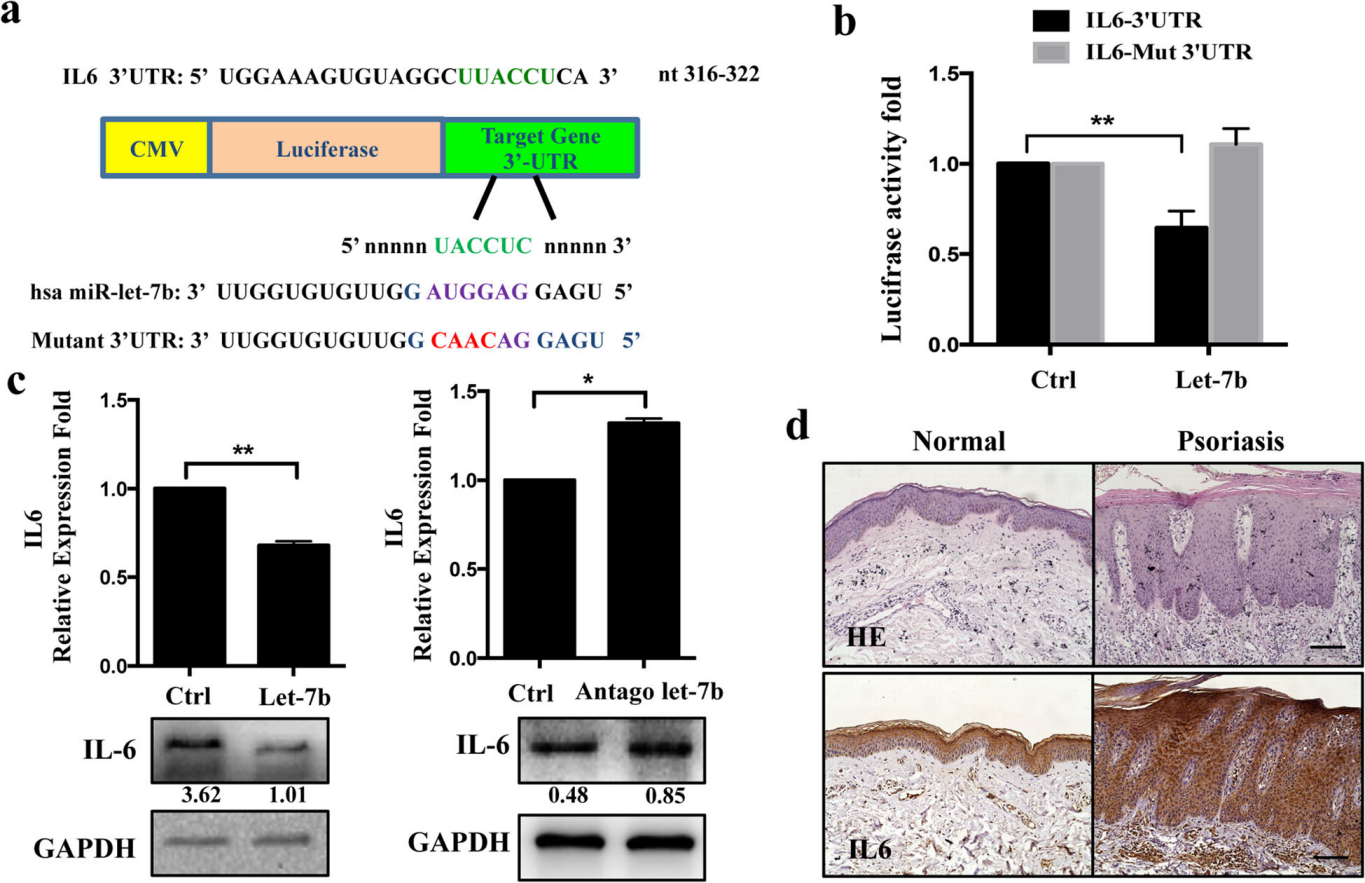
IL6 is a target of let-7b. **a** Schematic representation of chimeric luciferase constructs. The 3’ UTR sequences containing let-7b binding sites are shown (in green) for IL6. For the mutation of let-7b binding sites in the target 3’UTR, the bases in the seed sequence (in purple) were mutated to the sequence shown in red. Mutations were restricted to the conversion of AUGG to CAAC. **b** Insertion of let-7b target 3′-UTRs in a luciferase reporter gene constructs leads to reduced relative luciferase activity only in the presence of let-7b target sites. Mutant of let-7b target sequences of two sites in the IL6 3′-UTRs abolished the miRNA-mediated effect on luciferase activity. **c** Expression levels of IL6 in HaCaT were examined by qRT-PCR and western blotting after transfection. HaCaT were collected 48 h after transfection of let-7b mimic (left) or let-7b inhibitor (right) and scramble control sequences. **d** IL6 expression was analyzed in healthy (*n* = 4) and psoriasis lesional skin samples (n = 4) using immunohistochemistry, scale Bar = 100 mm

To gain insights into the molecular events associated with let-7b overexpression, we investigated how this miRNA affects keratinocyte differentiation. Using TargetScan 5.1 prediction software and miRanda, a set of mRNAs which encode proteins involved in cellular differentiation was identified. IL-6, a proinflammatory cytokine that acts on epithelial barrier function and keratinocyte differentiation [25], was identified as a potential target gene of let-7b with the predicted binding site at nucleotide positions 316 to 322 (Fig.4a). To validate whether IL-6 is a functional target of let-7b, a dual-luciferase reporter system was employed. We cloned the 3’UTR of IL-6 containing either the wild type or the mutated binding site of let-7b into the pMIR-Report vector, respectively, and co-transfected with let-7b mimics or controls into HaCaT. Data from luciferase assays show that over-expression of let-7b remarkably reduced luciferase activity regulated by the wild-type construct but not by the mutant IL-6 3’UTR construct in HaCaT (Fig. 4b). In addition, overexpression of let-7b caused a reduction of IL-6, whereas antagon of let-7b caused an IL-6 increase, both in mRNA and protein levels of HaCaT cells (Fig.4c). Moreover, IL-6 was found to be up-regulated in human psoriasis skin lesions compared with healthy skin (Fig.4d), suggesting that IL-6 might have a relationship with psoriasis presumably via let-7b expression levels.

**Fig. 5:**
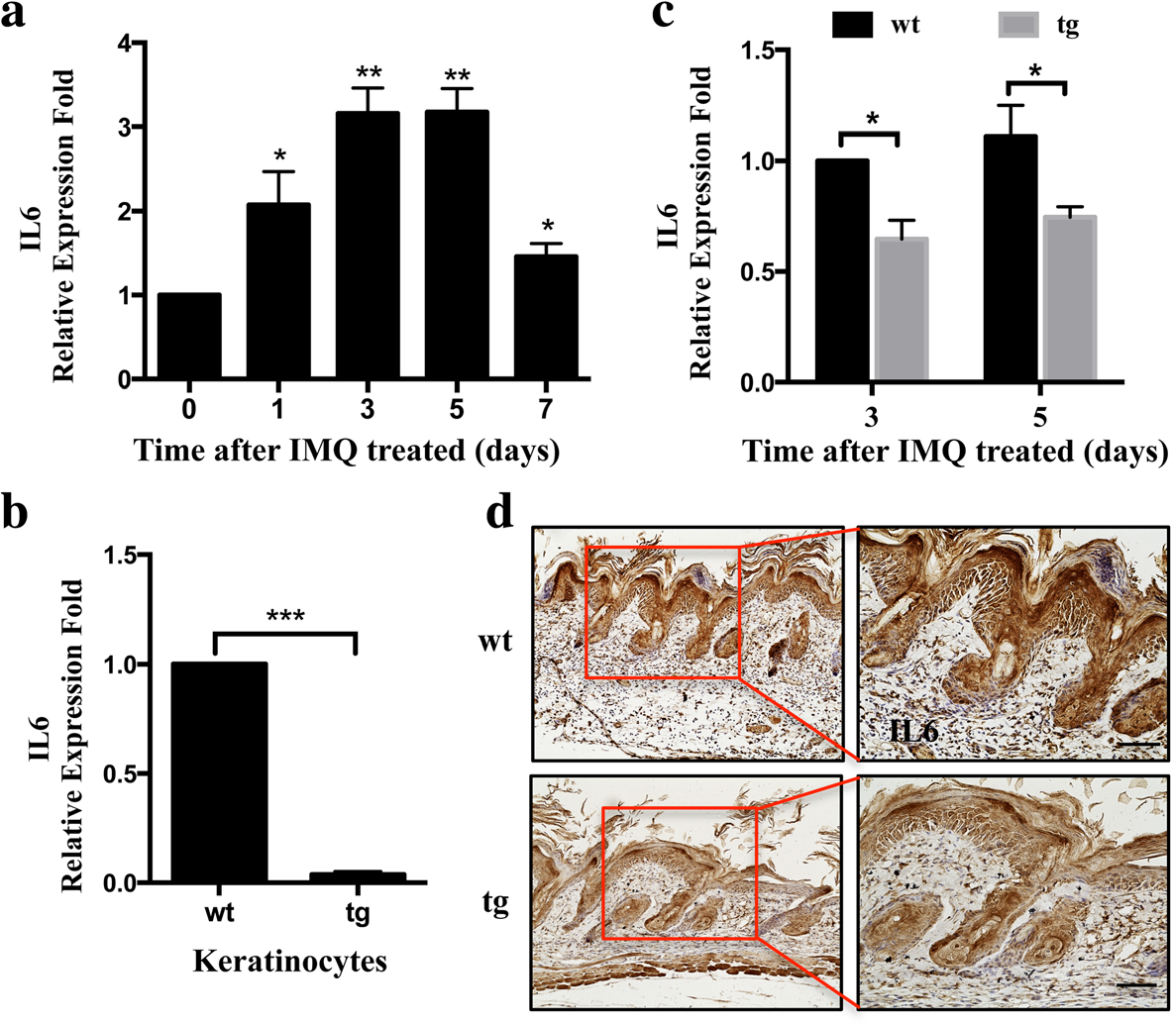
Let-7b suppresses expression of IL6 in a keratinocyte-specific let-7b transgenic mouse model. **a** qRT-PCR analysis of IL-6 expression in lesioned skin samples from IMQ-induced wild-type mouse model. **b** qRT-PCR revealed down-regulation of IL6 expression in primary keratinocytes from wild-type and transgenic mice. **c** qRT-PCR analysis revealed IL6 level of wild-type and transgenic mice treated with IMQ at days 3 and 5. **d** Immunohistochemical localization of IL6 protein 5 days after mice were treated with IMQ (*n* = 5). Histograms represent the average ± SD from three independent assays. *P < 0.05; **P < 0.01;***P < 0.01. Scale bars, 100 μm

In addition we found an increase in the expression levels of IL-6 by 2–3-fold at 1, 3, 5, 7 days after treatment with IMQ compared to healthy skin in wild-type mice (Fig. 5a). As expected, IL-6 was reduced in isolated primary keratinocytes at mRNA levels as shown by real-time PCR analyses (Fig. 5b). We further examined the expression of IL-6 from healthy skin and psoriasis lesions in transgenic mice and found a marked reduction of IL-6 by 30% compared with that of control littermates (Fig. 5c). In accordance with these results, immunohistochemistry demonstrated that IL-6 was mainly detected in the basal cell layer of the epidermis, and was expressed in both, let-7b^TG^ and wild-type mice psoriasis lesioned skin. However, IL-6 expression was significantly decreased in all epidermal layers in psoriasis lesions of let-7b^TG^ mice. From these findings, we conclude that IL-6 is a direct target of let-7b.

### Let-7b blocks ERK phosphorylation signaling

**Fig. 6:**
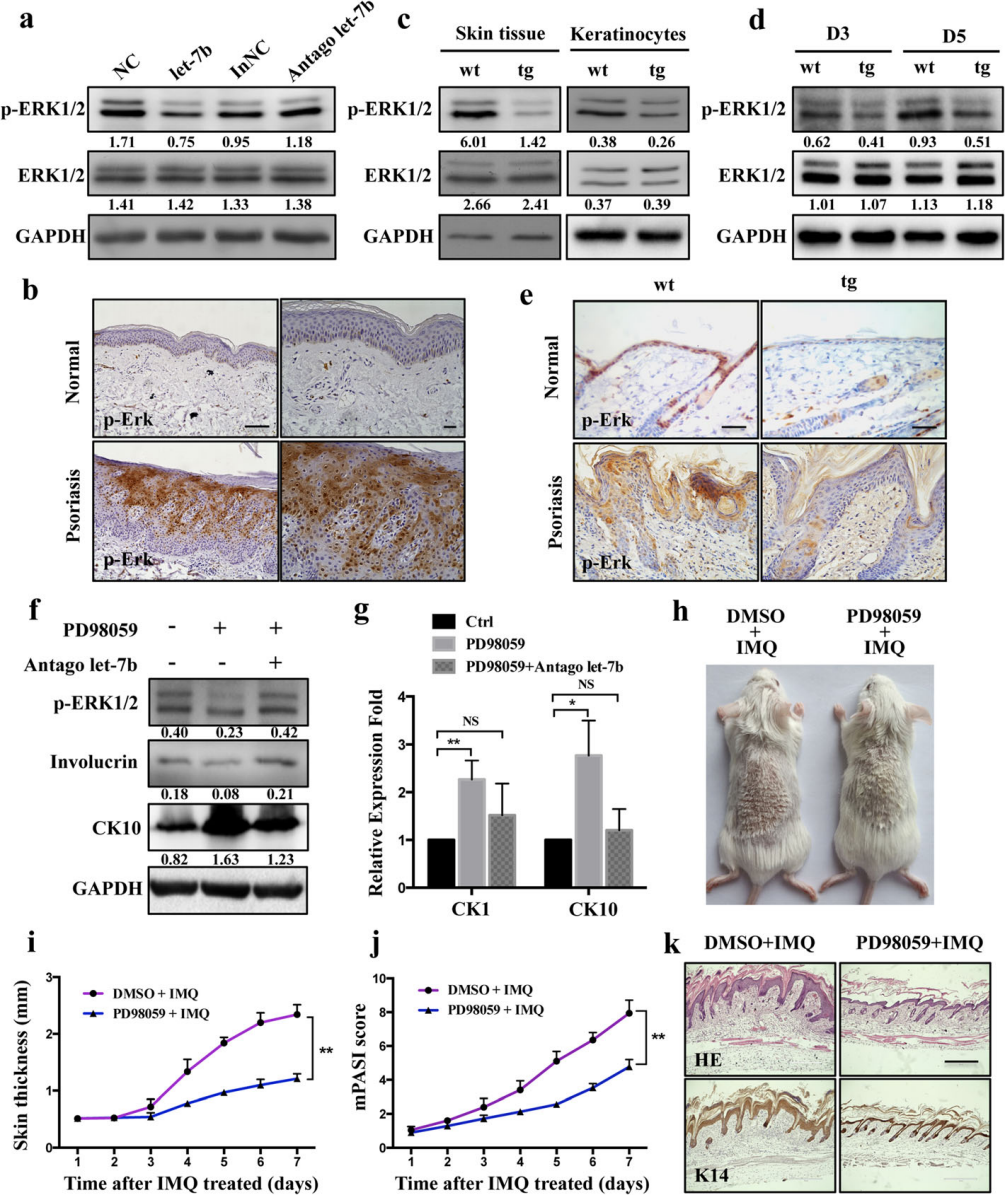
let-7b regulated the cell differentiation by reduces p-ERK1/2 signaling in vivo and in vitro. **a** HaCaT cells were transfected with let-7b mimics or with let-7b inhibitor (“antago let-7b”). Western blotting analysis of phosphorylated ERK1/2 (p-ERK1/2) and total-ERK (ERK1/2). **b** Immunohistochemistry analysis of p-ERK1/2 analyzed in healthy (n = 4) and psoriasis lesioned skin samples (n = 4). **c** Western blot analysis shows that p-ERK1/2 is decreased in skin tissue and in primary keratinocytes from keratinocyte-specific let-7b over-expression mice. Expression of total ERK shows no significant difference. Each protein sample results from pools of three mice with the same genotype. **d** Western blot analysis was performed to detect expression of p-ERK1/2 and total ERK1/2 in transgenic and control mice at days 3 and 5 after treatment with IMQ. **e** Immunofluorescent staining with p-ERK1/2 (*brown*) antibodies was performed in normal skin and after treatment with IMQ for 3 days in transgenic and control mice. Scale bars, 100 μm. **f**, **g** HaCaT cells were treated with MEK-ERK inhibitors (PD98059: 10 μM) and let-7b inhibitor. The differentiation markers was detected by Western blot (**f**) and RT-PCR (**g**). The results obtained analysis were done using t-test. *P < 0.05; **P < 0.01. (h) Phenotypic presentation of mouse back skin for wild-type treated with IMQ and ERK inhibitor (PD98059) for 7 days. The group treated with DMSO is control group. **i** Skin thickness was measured on the days indicated in the epidermis of mice treated with IMQ or vehicle as well as added PD98059 or DMSO. Symbols represent mean skin thickness ± s.e.m. for five to six mice per group. **j** mPASI reflecting the intensity of skin inflammation. Two-tailed unpaired Student’s t-test was performed for statistical analysis. Data are shown as means ± s.e.m. **k** Light microscopy examination of skin sections stained with H&E, or with keratin K14 antibody. Scale bar: 400 μm

It was reported that extracellular signal-regulated kinase (ERK1/2) phosphorylation is a crucial signaling pathway in the differentiation of mesenchymal stem cells and acute myeloid leukemia [26, 27], we sought to investigate the association between ERK1/2 signaling and let-7b expression in psoriasis development. Firstly, HaCaT was transfected with a let-7b mimic, which caused a decrease in p-ERK1/2 levels compared to control cells. Secondly, a slight induction of p-ERK1/2 was observed compared to control cells (“InNC”) when let-7b expression was inhibited (“Antago let-7b”, Fig. 6a). We next investigated p-ERK1/2 activation in the epidermis derived from lesioned skin of human psoriasis patients. Notably, by immunohistochemistry we found that levels of p-ERK1/2 were significantly increased in the epidermis of psoriatic lesions compared to healthy controls (Fig. 6b). In addition, Western blot showed that p-ERK expression markedly decreased in skin tissue of transgenic mice compared to wild-type mice. As expected, p-ERK was reduced in isolated primary keratinocytes at protein levels (Fig. 6c). In accordance with these results, phosphorylation of ERK1/2 was suppressed in normal skin and in 3 and 5 days after treatment with IMQ in wild-type and let-7b^TG^ mice (Fig. 6d, e). Further study we found cell differentiation was induced by treatment with inhibitors of MEK/ERK (PD98059), however, knockdown of let-7b can rescue the differentiation by regulation of phosphorylated ERK in HaCaT (Fig. 6f, g). At last, our study directly proved that blocking ERK signaling could alleviate psoriasis pathology in vivo using ERK inhibitor (PD98059) treatment (Fig. 6h). We demonstrated that PD98059 treatment in the epidermis resulted in a pronounced decrease in skin thickness in mice treated with IMQ (Fig. 6i), and a striking decrease of skin inflammation as reflected by mPASI (Fig. 6j). Microscopic examination of skin sections from mice revealed PD98059 could suppress skin thickening, parakeratosis and elongation of the dermal papillae (Fig. 6k).

## Discussion

Psoriasis is a chronic relapsing-remitting inflammatory skin disease that develops in genetically predisposed individuals after an unknown initial environmental, pathogenic or internal trigger. For decades, efforts have been made to explore the inflammation and keratinocytes proliferation or apoptosis role of miRNA in psoriasis while little is known about the roles of miRNAs in the development of psoriasis [28, 29]. In the present study we observed that let-7b directly acts on IL-6 and ERK signaling to affect keratinocyte differentiation. Notably, our findings confirm by in vitro and in vivo analyses in keratinocytes and in transgenic mice, respectively, that let-7b serves an essential role during psoriasis.

Previously, our group had established a keratinocyte-specific let-7b expression in a mouse model and explored a significant role let-7b in wound healing mediated migration in keratinocytes [17]. The sequential over-lapping phases during cutaneous wound healing closely resemble psoriasis lesional micro-environment. This led us to the question whether let-7b is instrumental in the pathogenesis of psoriasis. Among the numerous mouse models used for studying human psoriasis [30], the repetitive epicutaneous application of IMQ-containing imiquimod cream to the shaved mouse skin represents a versatile and efficient experimental regimen to study early events of human disease [31]. The results of our study confirm that let-7b^TG^ mice have less acanthosis and a reduced disease severity when treated with IMQ. It is well known that hyper-proliferation, altered differentiation of epidermal keratinocytes, and subepidermal angiogenesis are also observed in psoriatic lesions. However, previous study illustrated that let-7b does not affect keratinocyte proliferation and apoptosis, so we focused on the contribution of let-7b to cell differentiation in psoriasis.

The epidermis is a dynamic tissue mainly constituted by keratinocytes that undergo a tightly controlled differentiation program, moving from the basal to the suprabasal layers [32]. In the epidermal basal layer, cells express keratin 5 (CK5) and keratin 14 (CK14), while in cells of the upper (spinous) layers these are progressively down-regulated and replaced by keratin 1 (CK1) and keratin 10 (CK10) [33]. Thus, expression of KRT1 and KRT10 is the first indication of differentiation. In psoriasis skin that is characterized by reduced levels of differentiation markers (K10) and the balance between proliferation and differentiation is impaired [34]. During cornification, additional proteins such as involucrin,  loricrin and filaggrin are required to form corneodesmosomes and cornified envelopes [34]. Consistently, the results of the present study revealed an obvious decrease in CK10 production and increased Involucrin expression in skin biopsies of psoriasis patients, which is likely to be responsible for a significant suppression of keratinocyte differentiation. Interestingly, we observed a complete reversal of the expression of differentiation markers in let-7b^TG^ mice treated with IMQ, and we illustrate that let-7b affects keratinocyte differentiation in vitro.

With regard to the mechanism of let-7b in keratinocytes, potential target genes were determined. Among all potential target genes revealed by bioinformatics prediction software, IL-6 was selected as a suitable candidate for further analyses. It is known that stressed keratinocytes release IL-6 that recruits macrophages and neutrophils to sites of inflammation. Cytokines lead to abnormal keratinocyte maturation and activation of dendritic cells (DCs) [35]. Plasmacytoid DCs that are known to be involved in antiviral responses have been implicated in pathogenesis of psoriasis [21]. Although let-7b was reported to modulate inflammatory cytokines such as IL-6 in cholangiocarcinoma by short-term oxidative stress responses [36], the in vivo function of let-7b and the underlying mechanisms by which let-7b regulates cell differentiation in psoriasis is poorly understood. Here we identified the IL-6 gene as a direct target of let-7b to promote keratinocyte differentiation. Four separate bioinformatics tools predict that let-7b targets a sequence in the 3’UTR of IL-6 mRNA. In addition, IL-6 expression is significantly decreased in diseased epidermis of let-7b^TG^ mice compared to control mice treated with IMQ. Moreover, let-7b overexpression in HaCaT cells results in significantly decreased luciferase activity after transfection of HaCaT cells with a construct expressing the target sequence in the 3’UTR of IL-6. Lastly, the administration of a let-7b mimic amplifies let-7b function by suppressing IL-6 mRNA and protein levels in vivo. IL-6 has been shown to regulate inflammatory response and angiogenesis in a series of cell lines [37, 38]. The role of IL-6 in keratinocyte differentiation and in the pathogenesis of psoriasis was so far unknown; here we showed that IL-6 expression is augmented in the epidermis of lesioned skin from psoriasis patients. Overexpression as realized in let-7b^TG^ mice reduces IL-6 expression that leads to acanthosis and enhanced disease severity that is reduced by IMQ treatment. The data described above indicate that modulation of IL-6 expression in keratinocytes provide a basis for further studies to unravel the detailed mechanism of its differentiation regulation and a potential for future psoriasis therapies.

Furthermore, IL-6 mediated differentiation via signalling cascades, including ERK1/2, STAT3 and NF-κB signaling [39, 40]. The inhibition of ERK1/2 phosphorylation is associated with cell differentiation in certain cell lines [41]. In this study, we found that ERK1/2 phosphorylation was demonstrated in lesioned psoriatic skin compared to non-lesioned psoriatic skin, which is consistent with other reports and shows that active ERK1/2 suppresses differentiation of keratinocytes [42]. To our knowledge, no prior studies have addressed the possible direct intrinsic role of let-7b in keratinocyte differentiation and in the pathogenesis of psoriasis. Here we demonstrate that the overexpression of let-7b in transgenic mice leads to increased keratinocyte differentiation via ERK1/2 signaling, while inhibiting acanthosis and reducing disease severity in psoriasis mouse models.

## Conclusion

In conclusion, the present study has shown that let-7b overexpression increases epidermal differentiation and attenuates disease severity in mouse models of psoriasis. Let-7b directly targets IL-6, an essential cytokine regulating cell differentiation, which has been shown to be induced in the epidermis of lesioned skin from psoriasis patients. Our findings reveal a previously unknown mechanism for an homoeostatic let-7b-IL-6 axis whilst highlighting a critical role of ERK1/2 signaling in epidermal differentiation of psoriasis.

## Additional file

Additional file 1: **Figure S1.** Generation of keratinocytes specific let-7b transgenic mice. (a) The schematic diagram of generated the keratinocyte specific let-7b transgenic mice. (b) Let-7b expression was detected by real-time PCR in skin of wild-type and transgenic mice. (c) Relative let-7b expression level in keratinocyte of wild-type and transgenic mice as determined by microRNA qRT-PCR. The results obtained analysis was done using t-test. *P < 0.05; **P < 0.01 he results obtained analysis was done using t-test. *P < 0.05; **P < 0.01. **Table S1.** Primer Sequences. (PPTX 129 kb)

## Abbreviations

CK1: Keratin 1
CK10: Keratin 10
DCs: Dendritic cells
GAPDH: Glyceraldehyde-3-phosphate dehydrogenase
IGF2BP2: Insulin-like growth factor 2 mRNA-binding protein 2
IHC: Immunohistochemistry
IL-6: Interleukin 6
IMQ: Imiquimod cream
K14: Keratin 14
K5: Keratin 5
let-7b^TG^: Keratinocyte-specific let-7b overexpression mouse
miRNA: MicroRNA
MMP1: Matrix metalloproteinase-1
mPASI: Psoriasis Area and Severity Index of mouse
PD98059: Inhibitors of MEK/ERK
p-ERK1/2: Extracellular signal-regulated kinase phosphorylation
qPCR: Quantitative PCR
RT-PCR: Reverse transcription polymerase chain reaction
WT: Wild-type
