# Supplementary material for: MicroRNA let-7b inhibits keratinocyte differentiation by targeting IL-6 mediated ERK signaling in psoriasis
Source: Cell Commun Signal. 2018 Sep 15;16:58. doi: 10.1186/s12964-018-0271-9 (PMC6138911; doi:10.1186/s12964-018-0271-9)
Supplement: Supplementary file 1 — Figure S1. Generation of keratinocytes specific let-7b transgenic mice. (a) The schematic diagram of generated the keratinocyte specific let-7b transgenic mice. (b) Let-7b expression was detected by real-time PCR in skin of wild-type and transgenic mice. (c) Relative let-7b expression level in keratinocyte of wild-type and transgenic mice as determined by microRNA qRT-PCR. The results obtained analysis was done using t-test. *P < 0.05; **P < 0.01 he results obtained analysis was done using t-test. *P < 0.05; **P < 0.01. Table S1. Primer Sequences. (PPTX 129 kb) [file 12964_2018_271_MOESM1_ESM.pptx]

## Slide 1
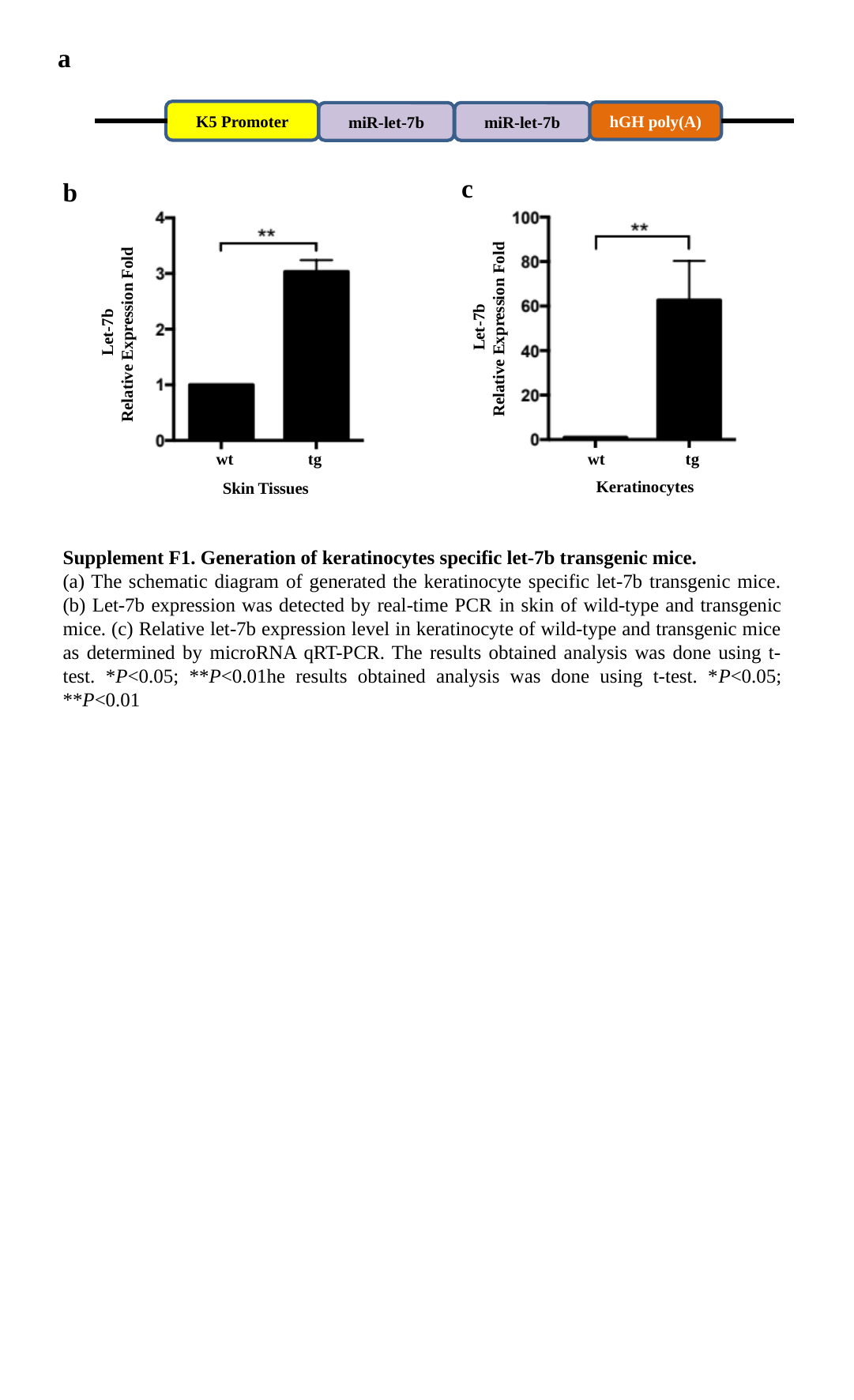

a
K5 Promoter
hGH poly(A)
miR-let-7b
miR-let-7b
c
b
Let-7b
Relative Expression Fold
Let-7b
Relative Expression Fold
wt
tg
wt
tg
Keratinocytes
Skin Tissues
Supplement F1. Generation of keratinocytes specific let-7b transgenic mice.
(a) The schematic diagram of generated the keratinocyte specific let-7b transgenic mice. (b) Let-7b expression was detected by real-time PCR in skin of wild-type and transgenic mice. (c) Relative let-7b expression level in keratinocyte of wild-type and transgenic mice as determined by microRNA qRT-PCR. The results obtained analysis was done using t-test. *P<0.05; **P<0.01he results obtained analysis was done using t-test. *P<0.05; **P<0.01

## Slide 2
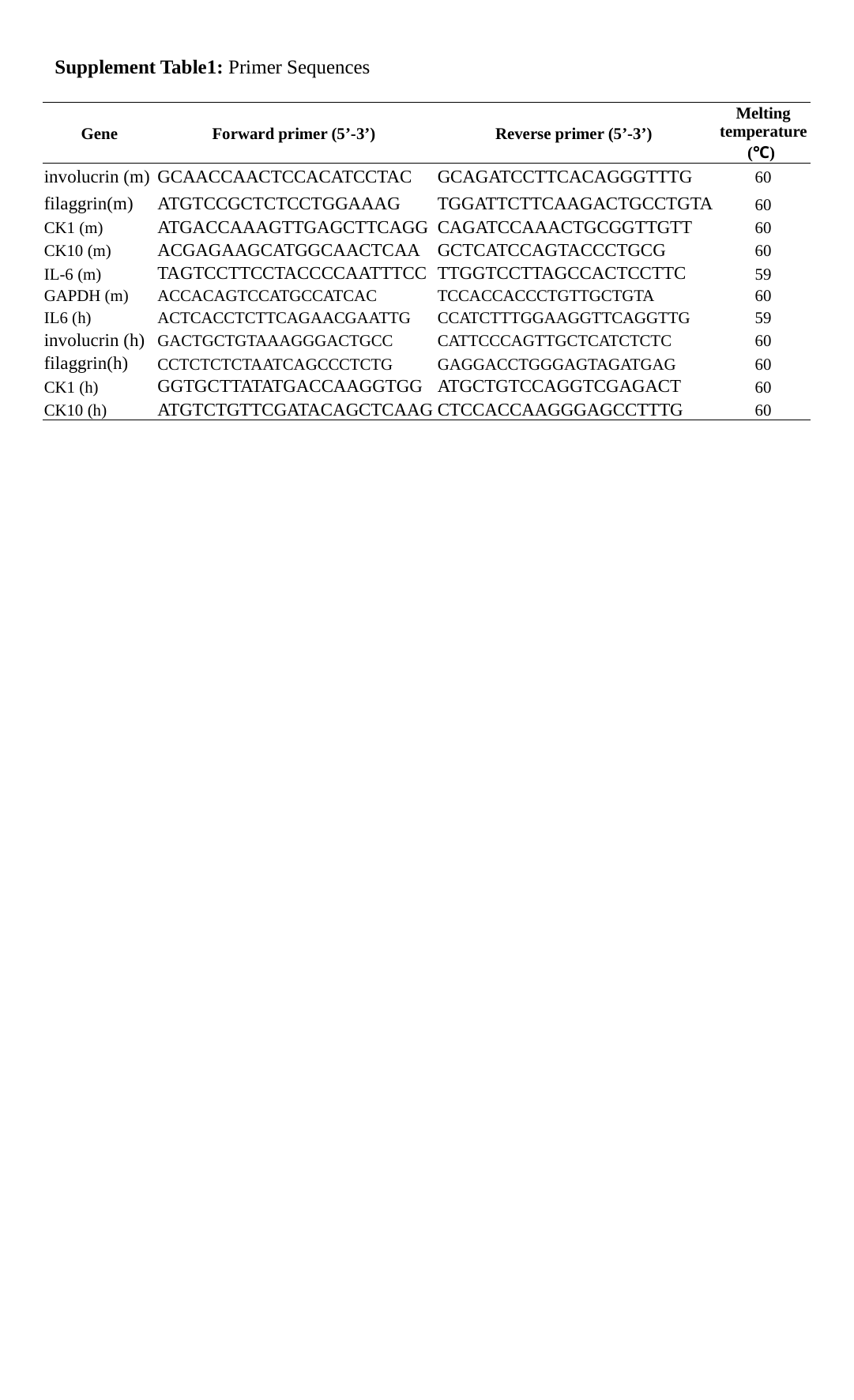

Supplement Table1: Primer Sequences
| Gene | Forward primer (5’-3’) | Reverse primer (5’-3’) | Melting temperature (℃) |
| --- | --- | --- | --- |
| involucrin (m) | GCAACCAACTCCACATCCTAC | GCAGATCCTTCACAGGGTTTG | 60 |
| filaggrin(m) | ATGTCCGCTCTCCTGGAAAG | TGGATTCTTCAAGACTGCCTGTA | 60 |
| CK1 (m) | ATGACCAAAGTTGAGCTTCAGG | CAGATCCAAACTGCGGTTGTT | 60 |
| CK10 (m) | ACGAGAAGCATGGCAACTCAA | GCTCATCCAGTACCCTGCG | 60 |
| IL-6 (m) | TAGTCCTTCCTACCCCAATTTCC | TTGGTCCTTAGCCACTCCTTC | 59 |
| GAPDH (m) | ACCACAGTCCATGCCATCAC | TCCACCACCCTGTTGCTGTA | 60 |
| IL6 (h) | ACTCACCTCTTCAGAACGAATTG | CCATCTTTGGAAGGTTCAGGTTG | 59 |
| involucrin (h) | GACTGCTGTAAAGGGACTGCC | CATTCCCAGTTGCTCATCTCTC | 60 |
| filaggrin(h) | CCTCTCTCTAATCAGCCCTCTG | GAGGACCTGGGAGTAGATGAG | 60 |
| CK1 (h) | GGTGCTTATATGACCAAGGTGG | ATGCTGTCCAGGTCGAGACT | 60 |
| CK10 (h) | ATGTCTGTTCGATACAGCTCAAG | CTCCACCAAGGGAGCCTTTG | 60 |
